# Bacteriophages and their implications on future biotechnology: a review

**DOI:** 10.1186/1743-422X-9-9

**Published:** 2012-01-10

**Authors:** Irshad Ul Haq, Waqas Nasir Chaudhry, Maha Nadeem Akhtar, Saadia Andleeb, Ishtiaq Qadri

**Affiliations:** 1 NUST Center of Virology & Immunology (NCVI), National University of Sciences & Technology (NUST), H-12, Islamabad 44000, Pakistan

**Keywords:** Bacteriophage, Phage therapy, Antibiotics, Vaccine, Biocontrol

## Abstract

Recently it has been recognized that bacteriophages, the natural predators of bacteria can be used efficiently in modern biotechnology. They have been proposed as alternatives to antibiotics for many antibiotic resistant bacterial strains. Phages can be used as biocontrol agents in agriculture and petroleum industry. Moreover phages are used as vehicles for vaccines both DNA and protein, for the detection of pathogenic bacterial strain, as display system for many proteins and antibodies. Bacteriophages are diverse group of viruses which are easily manipulated and therefore they have potential uses in biotechnology, research, and therapeutics. The aim of this review article is to enable the wide range of researchers, scientists, and biotechnologist who are putting phages into practice, to accelerate the progress and development in the field of biotechnology.

## Introduction

Bacteriophages are the most abundant entities on earth. These bacterial viruses have genetic material in the form of either DNA or RNA, encapsidated by a protein coat [1]. The capsid is attached to a tail which has fibers, used for attachments to receptors on bacterial cell surface. Most of the phages have polyhedral capsid except filamentous phages [2]. Phages infect bacteria and can propagate in two possible ways; lytic life cycle and lysogenic life cycle. When phages multiply vegetatively they kill their hosts and the life cycle is referred to as lytic life cycle. On the other hand some phages known as temperate phages can grow vegetatively and can integrate their genome into host chromosome replicating with the host for many generations [3]. If induction to some harsh conditions like ultraviolet (UV) radiations occurs then the prophage will escape via lysis of bacteria [3]. After the discovery of bacteriophages in early 20^th ^century many researchers thought about their (phages) potential of killing bacteria, which could undoubtedly make them possible therapeutic agents. But after World War II when antibiotics were discovered, this natural potential therapeutic agent got little attention and was only considered as a research tool for many years [1]. Bacteriophages have contributed a lot to the field of molecular biology and biotechnology and are still playing its part. Many mysteries of molecular biology are solved by bacteriophages. Today when everything is much more advanced than ever before, bacteriophages are getting enormous amount of attention due to their potential to be used as antibacterials, phage display systems, and vehicles for vaccines delivery [1]. They have also been used for diagnostic purposes (phage typing) as well [1]. In this review article all these applications have been summarized.

## Phage therapy

Phages as therapeutic agents in humans were first used in 1919 just when they were discovered [4]. Phage therapy started back in 1896 when Ernest Hankin first reported the existence of antibacterial activity against *Vibrio cholera *the causative agent of cholera which was considered one of the deadliest peril humans had faced [5]. In 1915, Frederick Twort hypothesized that antibacterial activity could be due to the virus (phage), but he did not pursue his discovery, therefore bacteriophages were discovered by Fe'lix d'He'relle in 1917 [5]. In 1925 d'He'relle reported treatment of plague (four types) by antiplague phages which drew attention towards phage therapy. Later on he visited India and worked on phage therapy of plague at the Haffkine Institute, Bombay (Mumbai) [6]. In west the concept of phage therapy died out in 1940 due to the emergence of antibiotics, but in former Soviet Union it was used and is still in practice. The Eliava institute in Tbilisi Georgia is considered the pioneer in this regard where phage therapy is extensively studied and applied [7].

West has remained reluctant to use phage therapy due to the unreliable early trials of phage therapy. But still phage therapy got attention in USA. William smith and his fellows reported the successful use of phages against *E.coli *in mice [8].

One of the reasons of the avoidance of the phage therapy in most of the western countries was unreliable and inconsistent results of many phage therapy trials. But today it is accepted that the main reason behind the failure was poor understanding of phage biology and some other issues like quality control during preparation of therapeutic stocks [9]. Phage therapy has been used in animals, plants, and humans with different degree of success. Phages have several potential advantages over antibiotics but at the same time it does have disadvantages as well.

The main advantage of phages is their specificity for target bacteria which reduces the damage to normal flora of the host greatly. The bacteria to be targeted must be identified first or otherwise a cocktail of phages should be used. Bacteriophages are self-limiting i.e. they require their hosts to be constantly growing; if the bacterial pathogens they are specific for are absent they will not persist for long enough [1]. Replication at the site of infection is another advantage of phages. They are safe with no or less side effects [10,11]. If bacteria become resistant to phages then phages do evolve naturally to infect the aforementioned resistant bacteria, hence minimizing the chances of bacterial escape, which scores another advantage of phage over antibiotics [10].

After their administration phages can dissipate very quickly throughout the body reaching almost every organ; but the immune system swiftly clears systemic phages which pose yet another problem to their acceptance as therapeutic agent [12,13].

One of the serious concerns about the use of phage therapy *in vivo *is a strong antibody response which would clear the phages more quickly and thus the use of phages for extended period of time would not be possible [1]. Other drawbacks of phages as therapeutic agents are their narrow host ranges, and the fact that phages are not always lytic under certain physiological conditions. During the preparation of phage stocks it must be ensured that phage preparations are free of bacteria and bacterial toxins in order to avoid secondary infections. But sterilizing phages could inactivate them. Phages may impart toxic properties to the bacteria resulting in virulence [5].

One way around is the use of phage lytic enzyme endolysin, rather than administering the whole virion [14-16]. Similarly genetically modified phages can be used, which will only deliver the DNA essential for making antibacterials that would be specific for the target bacteria [17].

At the moment it seems a bit far that phage therapy will replace antibiotics exclusively, but there is the hope that it will be used complementary to antibiotics especially for antibiotic resistant strains [1]. Phages will be much more reliable when used externally and where the immune system gives it a chance by favoring it to persist within the body for a little while [1].

## Phage display

**Figure 1:**
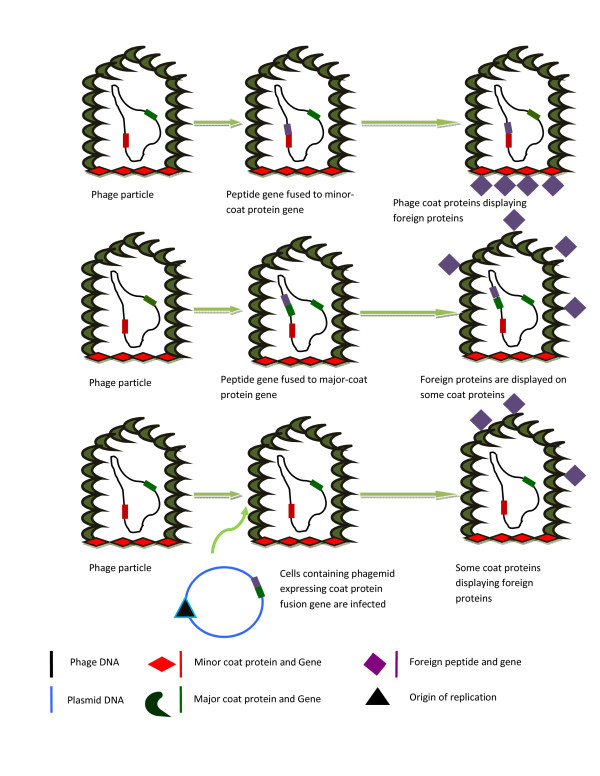
**Some methods that are used to fuse foreign peptides to the surface of phage**. Foreign peptides can be displayed on more than one phage coat proteins. Smaller foreign peptides are displayed in more numbers but it also depends on the type of antigen, coat protein and the phage. (a) The gene for a foreign peptide is directly fused to the minor coat-protein gene. The foreign antigen is displayed by all minor coat proteins. (b) Foreign peptide gene is attached to major coat protein gene while another copy of the gene (major coat proteins) is also present. Foreign protein is displayed on some major coat proteins. (c) Cells containing a phagemid (plasmid that have both plasmid and bacteriophage origin of replication) are infected with unchanged helper phage which then expresses the foreign peptide or protein. Foreign antigens are displayed by some coat proteins.

The concept of phage display was first introduced in 1985 [18] (Figure 1). Phage display is a molecular technique used for synthesizing polypeptides with novel characteristics. The DNA that encodes the polypeptide is fused with phage coat protein genes, and the desired protein is expressed on the surface of the phage particle [18,19]. For phage display filamentous phage M13 of *E.coli *is extensively used, other phages like lambda and T7 are also used in phage display system [20,21]. Phage display libraries can be used for the screening and isolation of peptides that are highly specific and which have affinity for target proteins. These peptides can be used in drug design as reagents for understanding molecular recognition and it also minimize mimics for receptors [19]. These peptides can be used as therapeutic agents by inhibiting receptor-ligand interaction or acting as agonist.

Moreover these proteins can be used for the detection of pathogens and agents that are considered to be a potential threat to the environment [22]. Directed evolution of proteins can be used to enhance the enzymatic activity and binding properties [23]. The active site of the enzyme is randomly altered and the activity of the enzyme is increased [1]. Phage display can also be varied by using phages to display the Fab antibody fragments library mostly on filamentous phage surfaces [24]. These libraries have many uses in research but one of the most important uses of it is in the treatment against cocaine addiction where phages are administered nasally and then ultimately they make their way to central nervous system (CNS). In central nervous system the displayed antibody binds to cocaine molecule and inhibits its action on brain [25]. Intensive and state of the art work done by many researchers have made phage display a phenomenal part of biotechnology. Amongst other applications phage antibodies have revolutionized the concept of therapeutic drugs and drug design [19]. Molecular evolution and protein-ligand interaction has been explained by phage display unambiguously [21].

## Phage typing

The specificity of phages for bacterial cells enables them to be used for the typing of bacterial strains and the detection of pathogenic bacteria [1]. Phage typing is also known as the use of sensitivity patterns to specific phages for precisely identifying the microbial strains. The sensitivity of the detection would be increased if the phages bound to bacteria are detected by specific antibodies [26]. For the detection of unknown bacterial strain its lawn is provided with different phages, and if the plaque (clear zones) appears then it means that the phage has grown and lysed the bacterial cell, making it easy to identify the specific bacterial strain [1]. There are certain other methods which can be employed to detect pathogenic bacteria such as the use of phages that can deliver reporter genes {e.g. lux} specifically [27] or using green fluorescent protein, [28] that would express after infection of bacteria. Similarly phages having a fluorescent dye covalently attached to their coats can be used to detect specific adsorption [29,30]. The detection of some of the released components such as adenylate kinase [31] after the specific lysis of bacteria and the use of antibodies and peptides that are displayed by phages can also be used, that will bind to toxins and bacterial pathogens specifically [22]. Dual phage technology is another application of phages in detection of bacteria, in which phages are used to detect the binding of antibodies to specific antigens [7]. Phage amplification assay can also be used to detect pathogenic bacteria [32]. The technique has most extensively been used for the detection of *Mycobacterium tuberculosis*, *E.coli, Pseudomonas, Salmonella, Listeria*, and *Campylobacter *species [33].

## Targeted gene delivery through Phages

Phages are the potential therapeutic gene delivery vehicles [33,34]. The rationale of using phages for targeted gene delivery is similar to that of using phages for DNA vaccines delivery in which the phage coat protects the DNA inside from degradation after it has been injected. But conceptually both are different. Phages ability to display foreign proteins on their surfaces enable them to target specific cell types which is a prerequisite for successful gene therapy [1]. Phage display and artificial covalent conjugation are used to display targeting and processing molecules on the surfaces of phages [35,36]. For the delivery of phages, targeting sequences such as fibroblast growth factor have been used to the cells having the appropriate receptors [37,38]. Enhancing the uptake and endosomal release of phages, proteins sequences such as penton base of adenovirus which mediates entry, attachment and endosomal release are used [39]. The protein transduction domain of human immunodeficiency virus (HIV) tat protein and the simian virus 40 (SV40) T antigen nuclear localization signal have also been used to enhance the uptake and nuclear targeting of phages like lambda that have been modified [40]. Other displayed peptides that can facilitate gene delivery via phages include integrin binding peptides which enhance binding and uptake [37] and DNA degradation reducing DNase II inhibitor [38]. To screen the ability of phages for targeting specific cells and tissues, phage display libraries have been used in mice many times and every time phages were found in specific tissues [41]. For instance isolating phages that target liver, mice were inoculated with phage display libraries and phages were isolated after extracting the livers [1]. Similar *in vitro *strategy is used for the isolation of phage displayed peptides that enhanced cytoplasmic uptake into mammalian cells [42]. So again phages proved themselves to be versatile by making it possible to target specific tissues either by screening phage display libraries randomly or by rational design [1].

## Phages as vehicles for vaccines delivery

**Figure 2:**
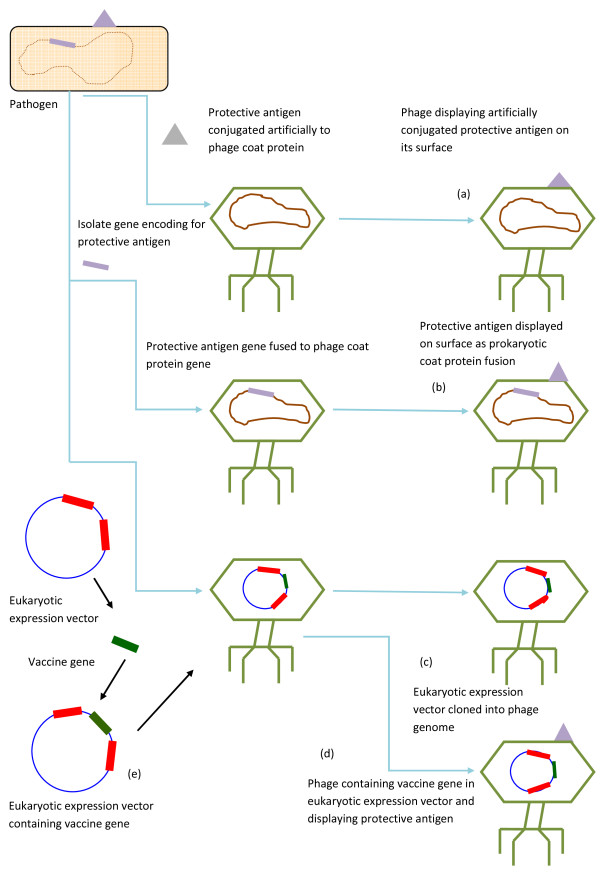
**Some examples of methods for vaccines delivery via phages**. (a) Host could be inoculated for phage-delivered protein vaccine. (b) As in (a) host is inoculated for phage-delivered protein vaccine but the protective antigen is expressed as prokaryotic coat protein fusion. (c) Inoculation of host for phage mediated DNA vaccination. (d) Host can be inoculated for hybrid phage vaccination, where in one construct protein and DNA vaccines are delivered through phage. (e) Host inoculation for standard DNA vaccination.

Phages have been used as vehicles for the delivery of vaccines (Figure 2). Phage particles can be used directly carrying the vaccine antigens expressed on their surfaces. But in case of DNA vaccines the sequences that are essential for the vaccine antigen synthesis are incorporated into the phage genome and the phage would then act as vehicle for the delivery of DNA vaccine [13]. Phage display can be used to construct phages that would display the specific antigenic peptide on their surfaces [1]. Phage display libraries can be screened with specific antiserum to detect novel antigens and mimetopes. Mimetopes are the peptides that mimic the antigenic properties and secondary structures of protective protein, lipid or carbohydrate, although having different primary structure [43,44]. Phage display libraries can also be screened against the serum of convalescents for the identification of potential vaccines against specific diseases [45]. There are some cases in which whole phage particles that displayed antigenic peptides have been used as vaccines in animal models [46,47].

Rather than transcriptional fusion to a coat protein, some substances can be artificially conjugated to the phage surface after growth, which will increase the range of displayed antigens [48]. Phages are considered to be natural immunostimulators [13,49] therefore an antigen that is presented on the phage coat protein would come 'ready conjugated' with a natural adjuvant activity, needing no separate protein purification and subsequent conjugation to a carrier protein before immunization. Recently it has been shown that unchanged phages can be used to deliver DNA vaccines more efficiently than standard plasmid DNA vaccination [13,50-52]. The gene for vaccine is cloned under the control of eukaryotic expression cassette in a lambda bacteriophage and purified phage particles are injected into the host. The coat protects DNA from degradation and as it acts as a virus-like particle it would target the vaccine to the antigen presenting cells [1]. When it was compared with the standard DNA vaccination, the antibody response was very much superior in mice [52] and rabbits [50]. Recently the possibility of producing a hybrid phage has been proposed, a DNA vaccine contained in phage particle under the eukaryotic promoter and a phage display variant of the same antigen is present on the phage surface [1]. Such a vaccine would efficiently target both humoral and cellular immune systems [13]. It can also be extended to the modification of surface of the phage vaccine by incorporating specific protein sequences to target particular immune cells types like galactose residues that will target galactose recognizing hepatic receptors in the liver [48]. Similarly dendritic [53] and langerhans cells could be targeted by isolating peptide from the phage display libraries [54].

## Phages as biocontrol and bacteriophage bioprocessing

Phages could be used as predators of pests (bacteria) found in association with plants, fungi or their products [55,56]. Phage mediated biocontrol of plant pathogens has successfully been attempted against *Xanthomonas pruni *associated bacterial spot of peaches to control infections of peaches, cabbage and peppers. Phages have also been used to control *Ralstonia solanacearum *of tobacco. They have been successfully employed against *Xanthomonas campestris *which cause spots on tomatoes. Similarly bacterial blotch of mushrooms caused by *Pseudomonas tolaasii *can be treated with phages [57]. Phages have also been considered as a means of controlling the bio fouling of thermal power plants condenser tubes [58]. Bacteriophages in bioprocessing are used to reduce the bacterial load in foods usually in the minimally processed foods to avoid cooking associated flavor or texture [59]. Controlling pathogens of fruits and vegetables is of much concern as these foods cannot be further processed that would kill any pathogen present. Control of pathogens via phages is a non-thermal intervention by which growth of *Salmonella *and *Campylobacter *on chicken skin [60]*Salmonella enteritidis *in cheese [61]*Listeria monocytogenes *on meat [62] and fresh cut fruit [63] is reduced. Extending the shelf life of animal products, phage bioprocessing could be used [64].

## Conclusion

Details given above give a glimpse of the large range of applications of phages in the field of biotechnology and medical science. The applications of phages range from the diagnosis of the disease, through phage typing, and its prevention (phage vaccine), to the treatment (phage therapy). There is the hope that phages could be useful to humans in many ways. By making a cock tail of phages it would become easy to treat a wide variety of bacterial infections that are otherwise resistant to the latest generations of antibiotics. A phage can be used individually to treat a bacterial infection by lysing the bacterial cell as it is having the lytic potential. At the same time the versatility of phages would allow us to use the antibodies against the bacteria that have been displayed on the phage surface. Similarly a protective antigen could be delivered as a DNA or phage display vaccine. So a mixture of phages that are modified genetically would be more helpful in addressing all these problems. Phages have also been good to cope with the food spoilage problem, and to treat the bacterial infection of plants and fruits.

There are some concerns about the use of phages. It includes the safety and efficacy issues, as well as immune response to the administered phages. Growth optimization and purification strategies of phages are also some issues needed to be addressed. Due to the rapid progress in the fields of biotechnology and molecular biology it is hoped that these entities (phages) which are present abundantly in the biosphere could answer many questions human beings are having.

## Abbreviations

HIV: Human immunodeficiency virus; CNS: Central nervous system; SV40: Simian virus 40.

## Competing interests

The authors declare that they have no competing interests.

## Authors' contributions

IUH surveyed the literature, collected the references concerned with this review and drafted the manuscript. MNA designed the figures. WNC, SA and IQ contributed in revising and editing the manuscript. All the authors read and approved the final manuscript.
